# The Role of Thiamine Pyrophosphate in Prevention of Cisplatin Ototoxicity in an Animal Model

**DOI:** 10.1155/2013/182694

**Published:** 2013-09-16

**Authors:** Ozan Kuduban, Cuneyt Kucur, Ebru Sener, Halis Suleyman, Fatih Akcay

**Affiliations:** ^1^Otolaryngology Department, Erzurum Training and Research Hospital, Cat Street, Yakutiye Avenue, 25341 Erzurum, Turkey; ^2^Otolaryngology Department, Evliya Celebi Training and Research Hospital, Dumlupinar University, Okmeydani Street, 43340 Kutahya, Turkey; ^3^Pathology Department, Erzurum Training and Research Hospital, Cat Street, Yakutiye Avenue, 25341 Erzurum, Turkey; ^4^Pharmacology Department, Erzurum Ataturk University Hospital, Yakutiye Avenue, 25345 Erzurum, Turkey; ^5^Biochemistry Department, Erzurum Ataturk University Hospital, Yakutiye Avenue, 25345 Erzurum, Turkey

## Abstract

*Objective*. The aim of this study was to evaluate the effectiveness of thiamine pyrophosphate against cisplatin-induced ototoxicity in guinea pigs. *Materials and Methods*. Healthy guinea pigs (*n* = 18) were randomly divided into three groups. Group 1 (*n* = 6) received an intraperitoneal injection of saline solution and cisplatin for 7 days, group 2 (*n* = 6) received an intraperitoneal injection of thiamine pyrophosphate and cisplatin for 7 days, and group 3 (*n* = 6) received only intraperitoneal injection of saline for 7 days. The animals in all groups were sacrificed under anesthesia, and their cochleas were harvested for morphological and biochemical observations. *Results*. In group 1, receiving only cisplatin, cochlear glutathione concentrations, superoxide dismutase, and glutathione peroxidase activities significantly decreased (*P* < 0.05) and malondialdehyde concentrations significantly increased (*P* < 0.05) compared to the control group. In group 2, receiving thiamine pyrophosphate and cisplatin, the concentrations of enzymes were near those of the control group. Microscopic examination showed that outer hair cells, spiral ganglion cells, and stria vascularis were preserved in group 2. *Conclusion*. Systemic administration of thiamine pyrophosphate yielded statistically significant protection to the cochlea of guinea pigs from cisplatin toxicity. Further experimental animal studies are essential to determine the appropriate indications of thiamine pyrophosphate before clinical use.

## 1. Introduction

Cisplatin is a mainstay chemotherapy drug in the treatment of a variety of solid tumors, notably testicular cancer. It is also used in the treatment of pediatric malignancies such as medulloblastoma and osteogenic sarcoma [1]. Cisplatin is cell cycle unspecific and is often used as a part in combination treatment. It has a toxic profile that is different from other important cytotoxic drugs. High doses cause nephrotoxicity, gastrointestinal toxicity, neurotoxicity, and ototoxicity, where the two latter side effects can be dose limiting even with modern preventive measures [2].

Inner ear toxicity is often a dose limiting side effect that hampers optimal cisplatin-based chemotherapy. It is normally manifested as a sensorineural hearing loss beginning in the high frequencies, successively progressing towards the speech frequency range [3]. It is often accompanied by transient or permanent tinnitus. Sometimes these problems can be severe, and ototoxicity and vestibular toxicity are usually irreversible [4].

Cisplatin ototoxicity has several characteristics. In man it is mainly evident in the basal turn of the cochlea as degeneration of the outer hair cells (OHCs) and to some extent the inner hair cells (IHCs) and associated nerves [5]. It has been shown that the toxic effect of cisplatin may result in a degeneration of the vestibular organs as well [6], although it is rarely diagnosed. Under experimental conditions, toxicity is normally manifested among the OHCs and in the stria vascularis. Histological alterations have also been observed among the spiral ganglion cells in the guinea pig [7].

Thiamine pyrophosphate (TPP) is the biologically active form of thiamine (vitamin B_1_), and it is an essential cofactor in all living systems. Microorganisms either synthesize TPP via *de novo* biosynthesis pathways or uptake exogenous thiamine from the environment via specific transporters. TPP plays a critical role in the carbohydrate and energy metabolism. In addition, TPP is involved in the *α*-oxidation of 3-methyl-branched and straight chain 2-hydroxy long chain fatty acids pathway functioning as coenzyme for peroxisomes. As a result TPP is a crucial cofactor for energy metabolism, antioxidation, and myelinization of nerve cells [21].

Continued high-dose cisplatin chemotherapy necessitates the investigation of strategies to decrease the dose-limiting ototoxicity. Lowering the dose intensity would not be a preferred option because this might reduce the efficacy of cisplatin. The aim of this study was to investigate the potential protective effect of TPP against the toxicity caused by cisplatin in the inner ear. This is the first publication, to our knowledge addressing the administration of TPP for cisplatin induced ototoxicity.

## 2. Materials and Methods

### 2.1. Chemicals

Cisplatin (cisplatinum, Ebewe, 0.5 mg/mL) was obtained from Liba Drug Company, Turkey. Thiamine pyrophosphate was obtained from SIGMA, Germany. All of the chemicals were of the highest quality commercially available.

### 2.2. Animals

Eighteen healthy adult male albino guinea pigs weighing 1200–1500 g (Erzurum Ataturk University Animal Laboratory, Turkey) were used in the current study. They had free access to water and food. The animals were kept under standard laboratory conditions, housed in a room at 20° ± 2°C temperature, and 12 h light/dark cycle. This study was performed with the approval from the Ataturk University Animal Care and Use Committee.

### 2.3. Experimental Design

Guinea pigs were randomly divided into three groups and treated as follows: group 1 (*n* = 6) received an intraperitoneal (IP) injection of saline solution and cisplatin (5 mg/kg) for 7 days, group 2 (*n* = 6) received a n IP injection of TPP (25 mg/kg) and cisplatin (5 mg/kg) for 7 days, and group 3 (*n* = 6) received only IP injection of saline for 7 days (employed as control group).

The animals in all groups were sacrificed under anesthesia (25 mg/kg thiopental sodium) and their cochleas were harvested for morphological and biochemical observations. All surgical procedures were performed under a dissecting microscope with sterilized instruments.

### 2.4. Biochemical Determination

The level of endogenous antioxidant glutathione (GSH), the activities of antioxidant enzymes glutathione peroxidase (GSH-Px) and superoxide dismutase (SOD), and the concentration of malondialdehyde (MDA), the end product of lipid peroxidation, were determined enzymatically with the techniques explained by the literature [8–11].

### 2.5. Histological Evaluation

In order to avoid cell destruction by autolysis or bacteria and to preserve tissue morphology and composition, the cochleas were fixed in 10% neutral buffered formalin for 24 h at +4C° temperatures. Subsequently, decalcification was achieved by submerging the samples in 10% EDTA at room temperature for 7 days. The specimens were then washed with tap water and fixed again in 10% neutral buffered formalin for 24 h.

Afterwards, the specimens were embedded in paraffin and then mounted in order to obtain mid-modiolar plane cuts. Sections of 5 *μ*m of thickness were collected on glass slides and stained with haematoxylin and eosin staining. Sections were examined using a light microscope (Olympus BX 51, Japan) and digital images were obtained by a digital camera (Olympus DP 71).

### 2.6. Statistical Analysis

The SPSS statistical software, version 13.0 was used for the statistical analysis. Significance of the difference between the groups and subgroups were analyzed using the one way ANOVA test and Fisher's post hoc least significant differences (LSD). A difference was deemed to be significant at *P* < 0.05.

## 3. Results

### 3.1. Biochemical Determination

There was a significant difference in level of GSH and MDA measurement and the activities of antioxidant enzymes GSH-Px and SOD among the groups (*P* < 0.05). Cochlear GSH concentrations, SOD and GSH-Px activities significantly decreased (*P* < 0.05) and MDA concentrations significantly increased (*P* < 0.05) in group 1, receiving only cisplatin, compared to the control group. In group 2, receiving TPP and cisplatin, the concentrations of GSH, MDA, and the activities of SOD and GSH-Px were near those of the control group. TPP restored the concentrations GSH and MDA and yielded statistically significant improvements in enzymatic activities of SOD and GSH-Px (*P* < 0.05) (Figure 4).

### 3.2. Histological Evaluation

**Figure 1:**
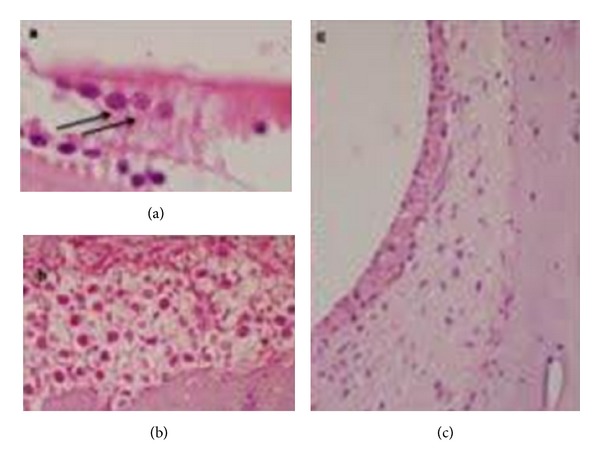
Normal microarchitecture of the organ of Corti, and (↑↑) OHCs can be seen (Figure 1(a), H&E; ×1000), spiral ganglion neurons (Figure 1(b), H&E; ×400), and stria vascularis without changes (Figure 1(c), H&E; ×400).

Samples obtained from the guinea pigs receiving only IP saline solution (group 3), revealed normal microarchitecture of the organ of Corti (Figure 1(a), H&E; ×1000), spiral ganglion neurons (Figure 1(b), H&E; ×400) and stria vascularis without changes (Figure 1(c), H&E; ×400).

**Figure 2:**
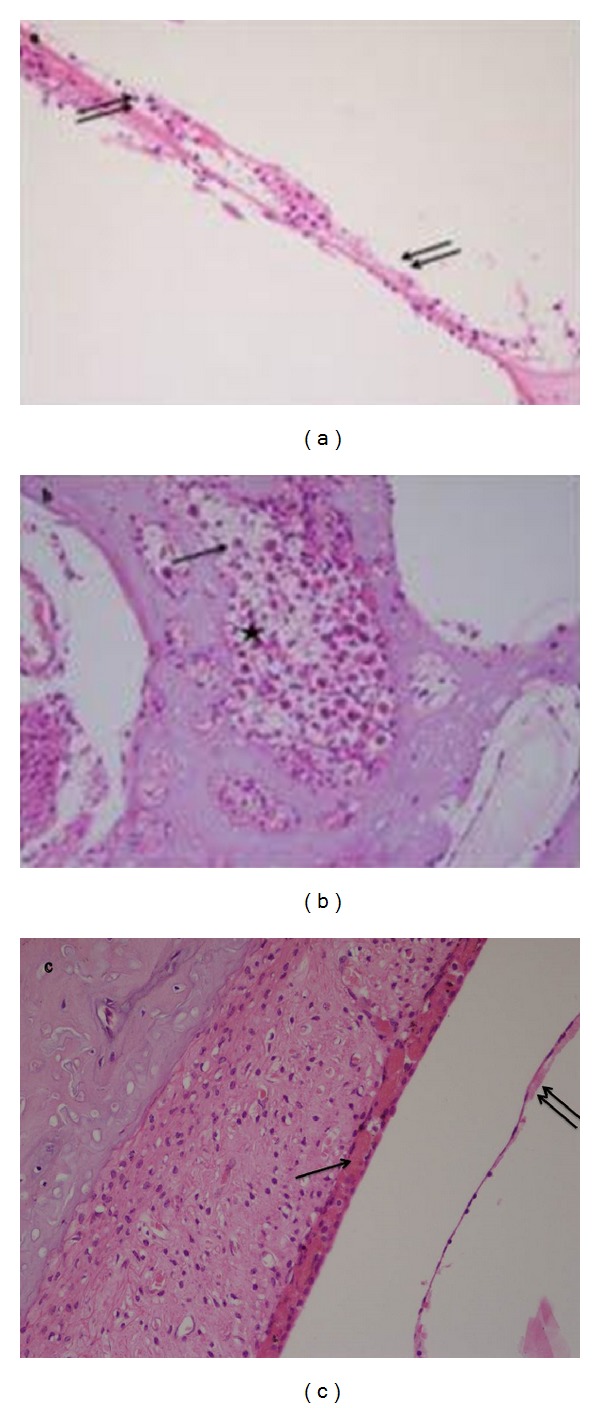
Showing extensive loss of the normal microarchitecture of the organ of Corti, severe destruction of the outer hair cells (↑↑). (b) Showing decreased number of spiral ganglion neurons with cell changes (star), such as vacuolation of the cytoplasm (↑). (c) Showing shrinkage of the intermediate cells (↑), edema of stria, and swelling of the epithelial cells of Reissner's membrane (↑↑).

Guinea pigs receiving the IP injection of cisplatin, we found an extensive loss of the normal microarchitecture of the organ of Corti; severe destruction of the outer hair cells (Figure 2(a)); scattered spiral ganglion neurons with cell changes, such as lack of nucleus, vacuolation of the cytoplasm, and partial detachment of the myelin sheath (Figure 2(b)); generalized change in the stria vascularis, including edema of stria and shrinkage of intermediate cells (Figure 2(c)).

**Figure 3:**
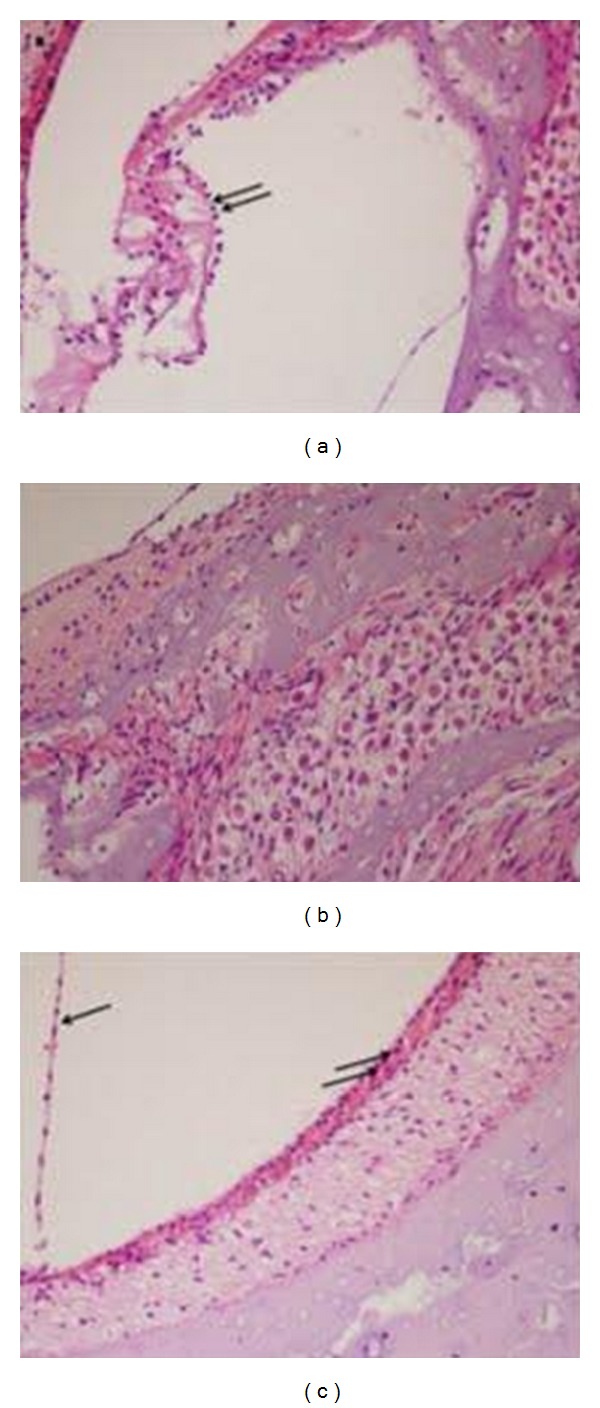
Showing the OHCs comparable to the control group (↑↑). (b) Showing spiral ganglion cells without destruction. (c) Showing apparently normal stria vascularis (↑↑) and Reissner's membrane (↑) as compared with the control group.

**Figure 4:**
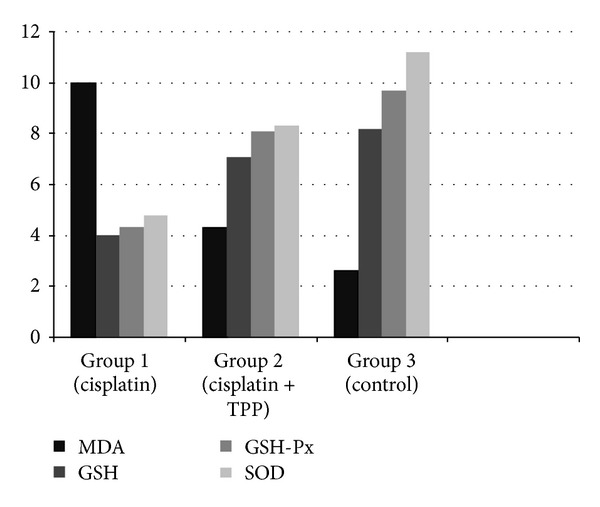
Showing comparison of mean levels between all groups.

On the other hand, guinea pigs receiving cisplatin and TPP exhibited preserved morphology of the tunnel of Corti and outer hair cells (Figure 3(a)), no destruction of spiral ganglion cells (Figure 3(b)), and no destruction of stria vascularis (Figure 3(c)).

## 4. Discussion

Cisplatin has a potent antitumor activity against several tumors, including germ cell, ovarian, lung, head, and neck cancers, but has dosage-limiting side effects (e.g., ototoxicity and neurotoxicity) [12]. Cisplatin ototoxicity leads to a bilateral and irreversible sensorineural hearing loss that is progressive from higher to the lower frequencies. It quickly binds DNA and proteins and thereby inhibits their functions. Once bound, cisplatin induces the generation of reactive oxygen species (ROS) that interfere with the antioxidant protection of the inner ear. This event may be a trigger for apoptosis and therefore decreases the number of cells in the cochlea [12, 13].

Cisplatin have three important tissue targets in the cochlea, including organ of Corti, spiral ganglion cells, and lateral wall (stria vascularis and spiral ligament). In guinea pigs that received consecutive cisplatin applications, destruction of outer hair cells and myelin sheath detachment of spiral ganglion cells were observed [14]. Furthermore depletion of glutathione and antioxidant enzymes (superoxide dismutase, glutathione peroxidase, and glutathione reductase) with an increase in malondialdehyde levels, an indicator of lipid peroxidation, were demonstrated in cochlear tissue samples from animals receiving cisplatin [15].

Numerous studies suggest that cisplatin induced hearing loss is mostly related with generation of ROS, initiating cascade oxidative mechanisms. Despite the presence of endogenous antioxidant molecules, including glutathione and the antioxidant enzymes, cisplatin induced oxidative stress can overwhelm these intrinsic defense mechanisms. Consequently, exogenous administrations of antioxidants have been the primary focus in the treatment of cisplatin induced ototoxicity [13].

At present, the only way to prevent cisplatin-induced ototoxicity is a limitation of the total dose per cycle, the cumulative dose, and the dose intensity [16, 17]. Obviously, this might reduce the efficacy of this cytotoxic agent. Therefore, there is a need to find effective protective drugs that prevent cisplatin-induced ototoxicity. Even though there have been studies with multiple otoprotective agents [18–20], none of these agents have been found to be unequivocally beneficial in preventing cisplatin ototoxicity, and no agent is currently recommended for routine use.

TPP is the biologically active form of thiamine (vitamin B_1_), upon entry into cells, thiamine is quickly converted to TPP that is the active substance. However, recent studies indicate that mammalian peroxisomes do contain TPP but that no pyrophosphorylation of thiamine occurs in these organelles, implying that thiamine must enter the peroxisome already pyrophosphorylated [21, 22]. That is why we preferred TPP rather than thiamine in this study. TPP as an antioxidant has been investigated in the treatment of several oxidative processes; however, it has not been previously evaluated for its potential protective effect against cisplatin ototoxicity. This present study showed that TPP was protected against cisplatin induced degeneration of cochlea, stria vascularis, and spiral ganglion cells. TPP also reduced the content of MDA and increased the cisplatin-mediated decrease in antioxidative enzymes (GSH-Px, SOD) and GSH levels. These results suggest that the antioxidant defense mechanisms of the cochlea were potentiated by this treatment.

In many forms of ototoxicity, pharmacological activation of intrinsic defense mechanisms could be helpful. Cisplatin induced ototoxicity is a special issue in that the ototoxic insult is predictable. It should be possible to administer protective agents at precisely timed intervals before the insult. The results of this present study suggest that TPP is beneficial in reduction of experimental cisplatin ototoxicity in guinea pigs, and it may be a potential candidate drug in human beings.

We have demonstrated the efficacy of systemic administration of thiamine pyrophosphate in the prevention of cisplatin induced ototoxicity using a guinea pig model. Further experimental animal studies are essential to determine the appropriate indications and dosages of TPP before clinical use.
